# Acceptability, Feasibility, and Effectiveness of a Worksite Intervention to Lower Cardiometabolic Risk in South Africa: Protocol

**DOI:** 10.3390/mps7020021

**Published:** 2024-03-01

**Authors:** Evonne Shanita Singh, Ashika Naicker, Shivneta Singh

**Affiliations:** Department of Food and Nutrition, Faculty of Applied Sciences, Durban University of Technology, 70 Steve Biko Road, Berea, Durban 4001, South Africa; ashikan@dut.ac.za (A.N.); 21616296@dut4life.ac.za (S.S.)

**Keywords:** intervention, worksite, cardiometabolic disease, diabetes, healthy, employee, South Africa

## Abstract

As an important way to translate cardiovascular disease prevention efforts, worksite intervention programs can be used to effectively facilitate healthy food choices, health education, and social support among employees, in a targeted approach to improve health outcomes and physical activity levels of employees. In this study, the effectiveness of a canteen and a behavioral intervention on cardiometabolic risk among prediabetic and prehypertensive employees at two multinational worksites in South Africa will be measured. This two-arm randomized controlled trial (RCT) will be structured to provide a six-week intervention at two multinational companies spread across eight worksites and will include a canteen and behavioral arm (CB) and a canteen only (CO) arm. Participants who are either prediabetic or prehypertensive will complete the baseline assessments, which will include anthropometry, a demographic and lifestyle survey, the global physical activity questionnaire (GPAQ) and the 24 h food recall. Participants will be randomized into the CO and the canteen and CB intervention groups. The CO group will receive six weeks of canteen intervention [changes to enable a healthy food environment], while the CB group will receive six weeks of canteen intervention along with a behavioral intervention. The behavioral intervention will include an intense six-week lifestyle program aligned to the Diabetes Prevention Program (DPP). This study will assess the added benefit of environmental-level changes aimed at lowering cardiometabolic risk in a low–middle-income country (LMIC) and has the potential for scale-up to other worksites in South Africa and globally.

## 1. Introduction

The leading causes of death in South Africa are tuberculosis (6%), diabetes (5.9%), cerebrovascular disease (5.1%), other forms of heart disease (5.1%), and Human Immunodeficiency Virus (HIV) (4.8%) amidst the slow progress in the treatment and management of non-communicable diseases (NCDs) within the prevailing double burden of disease [1]. In sub-Saharan Africa, rapid demographic, sociocultural, and economic transitions are driving the increase in the risk and prevalence of diabetes and other non-communicable diseases [2]. The actual extent of diabetes, cardiovascular risk factors, and macrovascular and microvascular complications in sub-Saharan Africa remain unknown [2].

Overweight and obesity, resulting from unhealthy diets characterised by an increase in the consumption of energy-dense food and beverages, coupled with a decrease in physical activity, are primary contributors to the non-communicable disease (NCD) burden in South Africa [3,4]. Concurrently, type 2 diabetes (T2D) and cardiometabolic diseases (CMDs), including cardiovascular diseases (CVDs), have moved to the second and third causes of death and disability, respectively, in South Africa [1]. In 2018, almost 60% of deaths in South Africa were attributed to NCDs, while communicable diseases accounted for under 30% of deaths [1]. Research indicates that lifestyle changes, including weight loss, increased physical activity, and improving the quality of an individual’s diet can prevent or delay diabetes and lower cardiometabolic risk factors like blood glucose, plasma lipids, and blood pressure [5,6,7]. However, the translation of lifestyle interventions into real-world settings is challenging. Moreover, the application of this knowledge to improve health outcomes in South Africa is not well established, especially for CMD primary prevention. To initiate change, it is important that enabling environments are created targeting modifiable risk factors to mitigate the impact of NCDs [8].

There is compelling evidence that worksite-based health interventions can have a positive influence on employees and worksites, overcoming barriers towards healthy lifestyle choices through the provision and leveraging of resources at a place and time where employees spend a large percent of their waking hours [9,10]. The environment in which an individual works can promote behavioral change and influence health-related outcomes. Additionally, worksites provide access to a relatively large group of adults and have the potential to be more sustainable due to social networks and peer support. Several intervention strategies to make worksite canteens healthier through environment modification have been well described [11,12,13]. The effect of these environmental-level initiatives has the potential to impact the entire worksite. However, the adoption of interventions depends on the feasibility, acceptability, ease of implementation, and integration at a worksite, while also considering available resources and support. Ideally, changes to the physical and food environment must be directed towards workers’ self-efficacy for behavioral change. By increasing self-efficacy, an environmentally focused approach may yield long-term effects beyond the intervention period and impact individuals’ eating behaviors and physical activity across multiple environments [14].

While evidence supports the effectiveness of lifestyle interventions at worksites, the success of these interventions relies heavily on the capacity to develop interventions grounded in an integrated framework of worksite governance, ultimately allowing for institutionalization for impact at scale. The first step to designing lifestyle interventions for worksites is to understand the worksite food and physical environment, to identify potential interventions, and to understand the existing barriers and facilitators of implementing those interventions in these settings. Then, a multicomponent worksite intervention will be designed and implemented that will consider the characteristics of the environment and will be informed by the views of workers, managers, and supervisors on the design, format, and delivery of the intervention. Our main objective is to assess the adoption (uptake of intervention), fidelity (degree of adherence to the planned intervention), acceptability (agreement and satisfaction with the intervention), feasibility (the contextual fit of an intervention), and effectiveness (the degree to which a targeted outcome is met) [15] of a worksite environmental intervention to reduce cardiometabolic risk in South Africa. As an important way to translate CVD prevention efforts, worksite interventions can facilitate healthy food choices, health education, and social support. In this study, we will measure the effectiveness of a canteen and behavioral (CB) intervention versus a canteen only (CO) intervention on cardiometabolic risk among employees at a worksite by evaluating the change in the number of participants meeting two or more cardiometabolic risk goals, namely reductions in blood pressure, triglycerides, and glycosylated hemoglobin (HbA1c) in each of the two arms of the intervention study.

This study had three overarching aims with specific objectives.

Aim 1. To recruit an optimal site for conducting a worksite intervention to lower cardiometabolic risk.

The specific objectives of aim 1 are as follows:

- To identify and recruit suitable worksites using a worksite characteristic checklist (Supplementary File S1).
- To recruit the worksite and attain gatekeeper permission to conduct the study at the worksite.
- To sign a memorandum of understanding with the worksite.

Aim 2. To develop an appropriate, acceptable, and feasible group of interventions to increase healthy eating at a worksite canteen and to improve physical activity within the worksite environment.

The specific objectives of aim 2 are as follows:

- To determine the capacity of the built environment through structured observations of the canteen and physical environment to offer healthy food and promote physical activity.
- To conduct semi-structured in-depth interviews (IDIs) with worksite and canteen managers and focus group discussions (FGDs) with employees to determine the appropriateness, acceptability, and feasibility of changes at the worksite.
- To determine the worksite readiness to implement change through the organizational readiness for implementing change (ORIC) questionnaire.
- To apply an intervention rating scale to canteen intervention components and a delivery strategy for implementation of the interventions, to worksite managers and canteen managers.
- To develop the canteen and a physical environmental intervention program using the findings from the formative study.
- To develop an intervention training manual and train canteen managers for the implementation of the food environmental intervention.

Aim 3. To implement and measure the effectiveness of a multi-component worksite intervention to reduce cardiometabolic risk.

The specific objectives of aim 3 are as follows:

- To measure the effectiveness of a canteen intervention on a composite score based upon improvement in cardiometabolic risk factors (2–3) with success defined by glycosylated hemoglobin (HbA1c) decrease ≥ 0.5%, a systolic blood pressure decrease ≥ 5 mmHg, and a decrease in plasma triglycerides ≥ 0.1 mmol/L [16,17,18]. We will compare the change in the participants’ score from the canteen intervention, from baseline to endline results.
- To measure the effectiveness of a CB intervention on a composite score based upon improvement in cardiometabolic risk factors (0–3) with success defined by HbA1c decrease ≥ 0.5%, a systolic blood pressure decrease ≥5 mm Hg, and a decrease in plasma triglycerides ≥ 0.1 mmol/L [16,17,18]. We will compare the change in the score from the CB group from baseline to endline results.
- To measure the effectiveness of a canteen intervention on diabetes risk by comparing the change in HbA1c of participants after the canteen intervention, from baseline to endline results.
- To measure the effectiveness of a CB intervention on diabetes risk by comparing the change in HbA1c of participants after the canteen and behavior intervention from baseline to endline results.
- To measure the combined effect of the CO and CB interventions on cardiometabolic risk by comparing the change in HbA1c, blood pressure, blood lipids, BMI, and WC of the participants from baseline to endline.
- To measure the effectiveness of a CO intervention on dietary behavior.
- To measure the effectiveness of a CB intervention on dietary behavior.

## 2. Research Plan and Methods

**Figure 1:**
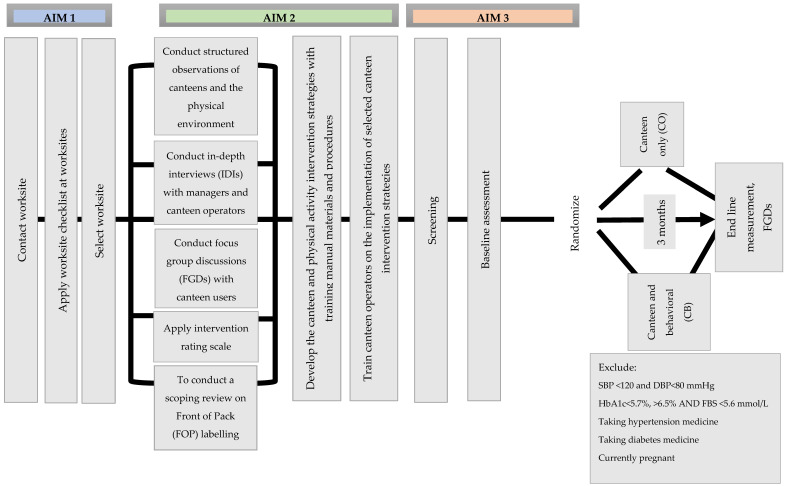
Overview of the study.

The South African Worksite Intervention Study is a three-phase intervention study (Figure 1). In the first phase, we will identify a suitable worksite for conducting the intervention study to lower cardiometabolic risk factors. The second phase consists of formative work to determine worksite capacity and readiness, as well as appropriateness, acceptability and feasibility of modifiable risk factors that would optimize contextual fit at the worksite, to reduce cardiometabolic impact. Feedback from the formative phase will be used to design training materials for the canteen staff and to advise lesson content for the lifestyle classes to be held during the intervention implementation phase. Phase three has multiple steps; the first step is a pre-screening step to recruit employees to participate in the study. The second step will be a screening step to recruit employees who fit the cardiometabolic risk profile. The third step will be the baseline participant data collection. In the fourth step, we will conduct an open-masked, single-blinded, two-arm simple randomized trial by allocating half of the participants to a CB intervention (lifestyle curriculum with physical activity) on the prevention of cardiometabolic risk, while the remaining participants will receive a CO intervention. Pre-post measures of the following cardiometabolic risk factors will be assessed: HbA1c, blood pressure, and lipids to determine intervention impact. The South African Worksite Intervention Study is based on a global model with similar research implementation at worksites in Nepal and India [16,17,18]. The schedule of enrolment and assessment for the South African Worksite Intervention Study is presented in Table 1.

### 2.1. AIM 1

We will apply a checklist at different worksites to identify the optimal industry partners in South Africa to conduct the intervention. We will evaluate the feasibility of conducting the intervention at each worksite. We will report results from the worksite observational checklist to identify the intervention worksite.

### 2.2. AIM 2

The second aim is to determine the capacity of the built environment through structured observations of the canteen and physical environment to offer healthy food and promote physical activity. The researcher will conduct structured observations using the worksite observation checklist (Supplementary File S2).

Semi-structured IDIs will be conducted with worksite managers and canteen operators to discuss the appropriateness, acceptability, and feasibility of changes at worksites and explore the perceptions, drivers, and barriers to healthy eating in the worksite environment. Purposive snowball sampling will be used to recruit worksite managers and canteen operators for the IDIs. Participants will include full-time staff in a management or supervisory position. A stakeholder map will also be used to identify participants.

The interviews will be scheduled at a time and place convenient to the participants, and time contributions from participants will be estimated as from 30 to 45 minutes. The IDIs for worksite managers and canteen operators will be conducted during a quieter period of the daily running of the canteen. Trained interviewers will use a semi-structured interview guide to conduct the interview. A digital recorder will be used to capture the audio of all interviews. The recordings will be used for quality checks and for transcription and translation, and for the iterative process of data collection.

Focus group discussions will be conducted to explore the perceptions, drivers, and barriers to healthy eating at the worksite. A FGD guide will be developed by the research team and will be reviewed for content and readability by the researchers. Flyers will be distributed strategically at the canteen sites, inviting employees interested in participating to contact the researcher. Purposive recruitment will be used to include information-rich employees representative of group diversity (different genders, age groups, and positions) at the worksite. The researcher will explain the study objectives, risks, benefits to participants, and time contributions from participants with an estimated time of one hour. Written consent will be obtained from participants. Privacy and confidentiality will be assured during and after the interview, and data will be de-identified. Trained moderators with matched gender to participants, to retain group homogeneity, will use the semi-structured FGD guide to conduct the FGDs. The FGD will be audio-recorded with a digital recorder, and a note-taker will transcribe the discussion. The recordings will be used for quality checks and for transcription and translation and will be deleted after one year of completion of the study.

An ORIC questionnaire will be administered at the worksite canteen during the lunch hour or online, through emails to employees, to determine the worksite readiness to implement change at the worksite [19] (Supplementary File S3). A list of email addresses will be obtained from the human resources department. The researcher will approach worksite managers and canteen operators who participated in the IDIs, to rate possible intervention components for the purpose of tailoring the evidence-based interventions and to identify the best way to deliver the interventions. The interventions will be rated by canteen managers or the manager of the worksite on a scale from one to five for the feasibility of implementing different components of the intervention, with one being impossible to implement and five being easy to implement.

Training manuals and materials will be developed, and canteen operators will be trained to implement optimal intervention strategies.

We will report the areas for intervention based on the responses to the rating scale. We will summarize results from the canteen and the physical environment observational checklist to identify emergent canteen and physical activity promotion strategies. The analytical strategy for the focus groups and the interviews used will assist us in understanding which intervention components are more likely to be implemented successfully, both in the physical and food environment. Inductive coding will be used to analyze the qualitative verbatim data from the IDIs and FGDs. This will allow findings like recurring, dominant, or significant themes to emerge. A codebook for each set of qualitative data will be created, tested for inter-coder reliability, and used to code the transcripts. Coding will enable us to identify emergent thematic elements that could inform the intervention. The emergent themes will be related to our research questions as to which canteen intervention and physical activity strategy are most likely to be implemented successfully.

### 2.3. AIM 3

The third aim is to measure the effectiveness of the program among participants by evaluating the change in the number of individuals reaching two or more of cardiometabolic risk goals, namely reductions of HbA1c (the primary outcome), blood pressure, triglycerides, and through changes in secondary outcomes, including rates of diabetes incidence and regression to normoglycemia and changes in anthropometry, lipids, and fasting glucose.

Design: Pre-post-test design among prediabetic and prehypertensive workers.

Screening and employee eligibility: All adult employees will be invited via letter or through an oral presentation of the letter at departmental general meetings. To identify individuals with prediabetes and prehypertension, we will conduct a two-step screening process. Employees will be eligible if (1) working full-time or on a long-term contract; (2) ≥18 y of age; (3) overweight or obese using the World Health Organization (WHO) cut-off points [20]; (4) have prediabetes (HbA1c of 5.7–6.4% [21] (not currently taking any diabetes medications); (5) have systolic blood pressure ≥ 120 mmHg or diastolic blood pressure ≥80 mmHg [22]; and (6) not pregnant or breastfeeding and with no history of heart disease, current serious illness, or conditions which would hinder participation in an unsupervised physical activity and diet change program. Exclusion criteria include (1) pregnant women; (2) on diabetes medication; (3) on hypertensive medication; or (4) screened individuals identified as diabetic or hypertensive. Screening part 1: Rapid Capillary Glucose (RCG) test (<5.6 mmol/L) and 2 blood pressure measurements (systolic <120 mmHg or diastolic <80 mmHg). Eligible participants will be invited to screening part 2: HbA1c of 5.7–6.4%.

Baseline assessment: Trained research assistants will assist the researchers in applying several questionnaires for the baseline data collection as follows:

Baseline socioeconomic and lifestyle questionnaire: will assess socioeconomic characteristics of participants, including lifestyle factors, smoking habits, and alcohol consumption.

Global physical activity questionnaire (GPAQ): will be used to calculate the metabolic equivalent of task (MET) minutes per week [23] at baseline.

Twenty-four-hour food recall: two interviewer-administered 24 h food recalls will be conducted within a week, to measure dietary intake at baseline. Energy and nutrient intakes will be calculated using the food composition tables for South Africa and analyzed using FoodFinder (FF3).

Anthropometry: Body weight, height, and waist circumference will be measured. The BMI will be calculated and classified in accordance with WHO cut-offs and standards [20] and the waist circumference will be classified according to the National Department of Health South Africa Demographic and Health Survey [21].

Laboratory: Baseline and endline blood samples will be analyzed for HbA1c, fasting glucose, low-density lipoprotein (LDL), high-density lipoprotein (HDL), triglyceride, and total cholesterol (TC), respectively. Evacuated blood collection tubes will be used to collect the blood samples, following an overnight fasting period of 8–10 h for the participants. A locally based biochemistry laboratory that follows the South Africa National Accreditation System (SANAS-accredited) methods of analysis will be used to conduct the laboratory procedures. The HbA1c will be measured using HPLC methodology (Biorad Variant Turbo 2), plasma glucose will be measured using enzymatic hexokinase method (Beckman AU480), LDL will be measured using enzymatic two-phase detergent reagent method (Beckman AU480), HDL using enzymatic color chromogen (Beckman AU480), triglyceride using enzymatic GPO-PAP (Beckman AU480), and TC using enzymatic cholesterol esterase method (Beckman AU).

### 2.4. Interventions

Step 1: Canteen interventions: will be available to all employees at participating worksites and will be based on findings from the in-depth interviews and focus group discussions which will be conducted during the formative phase of the research study. The canteen interventions will be implemented over a six-week period to help improve the nutritional value of food and beverage offerings within the worksite food environment (WFE).

Step 2: The behavioral intervention: half of the participants will be randomized to receive a CB, whereas the other half will receive CO intervention. The behavioral intervention will be comprised of a combination of intensive education sessions, goal-setting, and monitoring based on a validated worksite curriculum tailored to local needs. The curriculum includes 6 core weekly sessions followed by 2 weekly maintenance sessions (text messages). Each session will last one hour and will be facilitated by a lifestyle educator who will have a minimum of a master’s degree or a food and nutrition related postgraduate qualification. The lifestyle curriculum will cover the following subject matters: introduction to the diabetes and hypertension prevention program; tipping the kilojoule balance through understanding energy intake and output; eating a healthy diet and measuring portion sizes; identifying fats and carbohydrates and reducing salt intake; increasing physical activity; and stress management, alcohol and tobacco use, and ways to stay motivated. The range of infographics used during the canteen intervention will be included during the lessons. For the duration of the intervention, participants will be encouraged to keep food and activity diaries designed for the study. The maintenance period will be used to keep participants motivated and to instill long-term healthy behaviors. A maximum of 20 participants will be allocated per group. Participants will set at least two lifestyle change goals, such as consuming half of the total grains as whole grains, walking 30 minutes a day, reducing 7% of their body weight, and similar goals to achieve during the sessions based on their baseline CVD profile.

Follow-up: We will follow up with the participants at 3 months (i.e., at the end of the intervention). During the follow-up phase, fasting blood samples will be collected and analyzed for HbA1c, and lipid profile (HDL, LDL, TC, triglycerides). We will also re-administer the GPAQ and the 24 h food recall, and measure height, weight, waist circumference, and blood pressure.

Statistical Analysis: For our primary outcome, at 3 months, we will compare the proportion of participants who have attained two or more cardiometabolic risk factor reduction goals (HbA1c decrease ≥ 0.5%, systolic blood pressure decrease ≥ 5 mmHg, or triglycerides decrease ≥ 0.1 mmol/L) during the canteen intervention period and the baseline using generalized estimating equations. We will compare the proportion in the CB to CO, using the chi-square test.

#### 2.4.1. Data Analysis Plan

**Figure 2:**
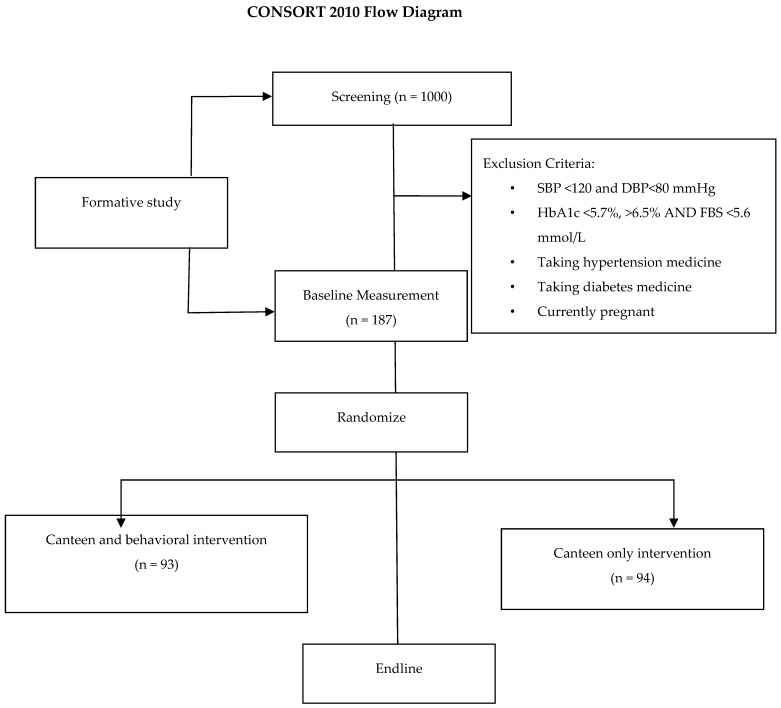
Planned flow of participants through the South African Worksite Intervention Study [24]. SBP: systolic blood pressure; DBP: diastolic blood pressure; HbA1c: glycosylated hemoglobin; FBS: fasting blood sugar.

The Statistical Package for Social Sciences (SPSS^®^ version 29IBM Corp, Armonk, NY, USA) will be used to analyze the data. The primary analysis will be focused on intention to treat. Descriptive statistics, including frequency tables, mean, median, mode, and standard deviation will be used to summarize, highlight, and describe core characteristics of the pre-screening, BL, and EL data. Inferential statistics will be used to test the hypothesis/aims. The chi-square goodness-of-fit-test will be used to check categorical data sets for distribution of responses and findings to assess if any responses and findings presented more or less frequently than others. The quantitative data analysis will follow the Consolidated Standards of Reporting Trials (CONSORT) guidelines (Figure 2). Flowcharts will include the number of participants projected for each stage, including the number to be screened, eligible for randomization, and analysis for the primary outcomes. Screening: for this study, 350 participants are required. A total of 1000 participants will be screened to determine eligibility and to obtain the sample size and to account for dropouts.

#### 2.4.2. Effectiveness of the Canteen Intervention

At the 3-month follow-up, we will evaluate the effectiveness of the canteen intervention by comparing the percentage of participants who have successfully attained at least two of their cardiometabolic risk reduction objectives during the canteen intervention phase at baseline with those who achieved the same during the endline phase.

#### 2.4.3. Effectiveness of the Canteen and Behavioral Intervention

At the 3-month follow-up, we will gauge the effectiveness of the behavioral CB intervention by comparing the percentage of participants in the CO who have met two or more of their cardiometabolic risk reduction goals to those in the CB group (as randomization may not adequately balance baseline characteristics between the intervention and control groups, this comparison becomes particularly relevant).

### 2.5. Process Outcomes

Measuring the following implementation outcomes will help us better understand the implementation processes of the intervention and will provide valuable data on the success and failures of the intervention, in addition to its effectiveness:

1. Program adoption will be measured by quantifying participation in the program and by tracking success in achieving the goals of the lifestyle education program (7% weight loss, at least 30 minutes of physical activity, and pre-post dietary improvements). Program adoption will be measured by quantifying workers’ participation by calculating the percentages of total employees agreeing to the screening, and percentages of eligible employees agreeing to participate further in the intervention study.
2. Program fidelity will be determined by measuring changes in the worksite environment, and management support for the program. To assess compliance with the recommended dietary interventions (i.e., provide healthy food options in the canteen), every month in the intervention period, a member of the study team not affiliated with the worksite will do a random audit.
3. Program feasibility will be measured by quantifying the changes in sales of healthy and unhealthy foods and beverages at the canteen from baseline to follow-up.
4. Program adoption and acceptability will be assessed through FGDs with employees (lifestyle participants and lifestyle dropouts), managers, and non-eligible participants on worksite environmental changes.

To measure the value and return on investment of the intervention for employers, we will assess program cost and cost-effectiveness and changes in staff productivity, absenteeism, health status, and quality of life:

(a) Estimate total cost to deliver the intervention: the average cost (fixed and variable) to deliver the lifestyle intervention program at the worksite will be estimated. Cost data from all components of the worksite intervention will be collected:
  1. Delivery of the lifestyle education program, which includes labor costs for lifestyle educator, classes, and education materials;
  2. Changing the work environment, which includes the costs of modifying the worksite food environment.
(b) Staff productivity (absenteeism, presenteeism) and indirect costs: absenteeism will be calculated based on worksite attendance records. To determine presenteeism, we will administer a questionnaire that captures the performance of employees (specific markers of productivity for each worksite and employee function). We will calculate indirect costs (monetary value of lost productivity) using the human capital approach (multiplying combined days lost to absenteeism plus presenteeism by salary of different occupational roles).
(c) Health status and healthcare utilization of employees: employees participating in the research will use the research questionnaires relating to health status, hospitalizations, and medication use, to self-report their health status.

### 2.6. Sample Size

One hundred and eighty-seven participants will form the consenting target population and will be randomly assigned to one of the two intervention groups, namely CB or CO. Assuming an alpha level of 0.05 and a margin of error of 0.05, the minimum sample size from a population of 187 needed to identify significant improvements in at least 2 out of 3 measures from baseline to 3 months is approximately 65 (*p* = 0.143) in the CO group and 67 (*p* = 0.286) in the CB group. To identify a significant change in HbA1c from baseline to 3 months, and assuming a value for alpha of 0.05, sample sizes of approximately 80 will be needed to identify clinically significant changes with a power of 80% for the CB and CO groups.

### 2.7. Minimization of Contamination

During the behavioral intervention, we will closely observe and assess the risk and extent of cross-contamination at the participant level. Participants will receive specific instructions not to share any information related to the study and not to offer any guidance regarding the intervention to any colleagues at their worksite, except those who have been designated to receive the behavioral intervention. Furthermore, we will gauge the potential contamination by inquiring whether participants assigned to the CO group have obtained any information or guidance relating to dietary and lifestyle changes from their peers in the CB group. If such contamination is identified, we will document the name and contact details of the peers involved. If significant contamination is detected, we will make appropriate adjustments when estimating the intervention’s impact.

### 2.8. Ethics and Governance

The researchers will approach worksites (through a gatekeeper letter) to participate in the study. Once the worksite is identified, employees will be briefed about the study through an information letter and meetings. For phase two of the study, announcements will be made using flyers to recruit participants at key points at the worksite. An organogram will be requested from human resources, and participants will be approached to participate with the necessary information letter and informed consent. Willing employees will be screened for eligibility after receiving a letter of information describing the study. All eligible, willing participants will complete an informed consent form. Confidential data will be archived for five years in the departmental archives, and then paper materials will be shredded and audio deleted. The results will be made available to employees during screening and at various follow-ups. For the blood draw, a SANAS-accredited laboratory will be used. Participants will have access to their screening data. They can make informed decisions from this data about whether they would like to continue participating in the study.

### 2.9. Exploitation, Dissemination, and Expected Impact

The impact of this study is multifaceted. The worksite-based program addresses an issue of major public health importance. The protocol focuses on both individual and environmental changes and includes a multi-pronged intervention strategy. Integrated innovation theory posits that for an intervention to be successful in complex situations, it must integrate scientific/technological, social, and business innovations. This randomized control study fulfils this requirement. It delivers scientific innovations (canteen interventions and lifestyle education) with social innovations such as trained a peer health champion to deliver the lifestyle education, and business innovation, such as worksite stakeholder commitment and researchers to oversee program fidelity, improvement of the worksite health environment, and evaluation of the intervention. If the study demonstrates feasibility, acceptability, effectiveness, and cost-effectiveness at the worksite/s, the results will be used to advise strategies for establishing and maintaining comparable lifestyle programs at other worksites in South Africa.

Current evidence indicates that health and wellness programs at worksites provide numerous benefits with respect to altering cardiovascular risk factor profiles. Implementing health programs at worksites allows for the opportunity to continually engage adults in positive and sustainable lifestyle choices. This study will explore the impact of an environmental intervention to decrease cardiometabolic risks and will estimate the additional benefit of an evidence-based individual-level diet intervention on cardiometabolic risks. If this study shows a significant impact, a scaled-up approach might result in a meaningful reduction in the burden of cardiovascular disease through environmental and individual-level prevention programs. The lessons learnt may also be replicated at similar worksites in South Africa and translated to similar settings globally. The project at an institutional level will boost human capital development (staff and student), create new knowledge, and boost research outputs. This support network will have a knowledge spillover effect on all local researchers (staff and students) working on this project. Ultimately, this research will identify whether a worksite intervention is a viable intervention for the prevention of NCDs in the South African setting and will add to the knowledge base of acceptable and viable prevention strategies for NCDs.

## Figures and Tables

**Table 1:** The schedule of enrolment and assessments for the South African Worksite Intervention Study.

	Screening 1	Screening 2	Baseline	Endline
Baseline: demographic information, health history			X	
Anthropometry				
Height			X	
Weight			X	X
Waist circumference			X	X
Blood pressure	X		X	X
Lifestyle factors			X	
24 h food recall			X	X
Global physical activity questionnaire			X	X
Smoking and drinking habits			X	
Lifestyle behavior			X	
Laboratory				
Glycated hemoglobin		X		X
Fasting blood sugar	X			
High density lipoprotein			X	X
Low density lipoprotein			X	X
Triglyceride			X	X
Total cholesterol			X	X

## Data Availability

Not applicable.

## Supplementary Materials

The following supporting information can be downloaded at: https://www.mdpi.com/article/10.3390/mps7020021/s1, Supplementary File S1: Worksite characteristics checklist; Supplementary File S2: Worksite observation checklist; Supplementary File S3: Organizational readiness for implementing change (ORIC) questionnaire.
