# Supplementary material for: Acceptability, Feasibility, and Effectiveness of a Worksite Intervention to Lower Cardiometabolic Risk in South Africa: Protocol
Source: Methods Protoc. 2024 Mar 1;7(2):21. doi: 10.3390/mps7020021 (PMC10961816; doi:10.3390/mps7020021)
Supplement: Supplementary file 1 [file mps-07-00021-s001.zip › mps-2865801-supplementary.pdf]

SUPPLEMENTARY FILE S1: WORKSITE CHARACTERISTICS CHECKLIST

Name of Assessor: Date: Start Time:

Name of worksite: Location: Number of sites:

Type of worksite: Blue Collar White Collar

Please tick Yes or No

	**Characteristics**	**Yes**	**No**	**Comments**
**1**	**Organizational size**: At least 1000 permanent, contract employees (will work for 2 years or more)			
**2**	**Gender**: At least 100 women permanent, contract employees			
**3**	**Canteen**: At least one at the site of the organization that serves two or more meals per day (breakfast, lunch, snack, dinner, etc.)			
**4**	**Canteen characteristics:** Most employees (at least 50- 75%) should eat one or more meals in the canteen at least most days a week (4 days or more)			
**5**	**Canteen intervention:** No or minimal intervention to improve food quality in the canteen			
**6**	**Willingness of management:** Willingness and commitment of the organization head/representative or management to make changes in canteen to improve food quality			
**7**	**Willingness of cafeteria staff:** Willingness and commitment of the canteen head/staff or management to make changes in canteen to improve food quality			
**8**	**Lifestyle intervention**: Willingness of the organization head/representative or management to allow employees to participate in lifestyle intervention (an hour per week for 6 weeks)			

SUPPLEMENTARY FILE S2: WORKSITE OBSERVATIONAL CHECKLIST

Environmental assessment for physical activity

Worksite:

Name of Assessor: Date: Start Time

SN		Yes	No	Comments
**1**	**GENERAL LOCATION**<br>The nearest intersection (crossroad) of the worksite			
1.1	Do you characterize this area as a city center? (**Take a picture**)			
1.2	Do you characterize this area as an industrial township? (**Take a picture**)			
**2**	**PEDESTRIAN INFRASTRUCTURE (Outside the worksite)**			
2.1	Are there footpaths (pavement) present in the area? (**Take a picture**)			
2.2	Is the pavement (footpath) wide enough for people to walk in both directions? **(Take a picture)**			
2.3	Are there vendors on the pavement (footpath)? (**Take a picture**)			
2.4	Is there a buffer between the pavement and the road? (e.g., a grass strip, trees, on-street parking**) (Take a picture)**			
2.5	Are ramps present at the intersections and driveways?			
2.6	Are sidewalks in good condition?			
2.7	Are there safe places to cross the streets? **(Take a picture)**			
**3**	**BICYCLING INFRASTRUCTURE**			
3.1	Are there dedicated lanes or marks for cycling? **(Take a picture)**			
3.2	Is it safe to cycle on the road?			
3.3	Is traffic flow fast and aggressive?			
3.4	Is the nearest bus stop or public transit within 10 minutes walking distance from the worksite?			
3.5	Generally, does the area feel safe and secure for pedestrians?			
**4**	**WORKSITE PREMISES**			
4.1	Is there bicycle storage space? (Take a picture)			
4.2	Is there a walking track on the worksite premises? (Take a picture)			

4.3	How many buildings are there on the premises? (Take a picture)			
4.4	How many floors are there in the tallest building? (Take a picture)			
**5**	**WORKSITE BUILDING**			
5.1	Are stairs visible from the main entrance? (Take a picture)			
5.2	Are stairs accessible before the elevator/escalator?			
5.3	Are stairs encountered before the elevator/escalator? **(Take a picture)**			
5.4	Is directional signage provided from the entrances to indicate location of stairs? **(Take a picture)**			
5.5	Is directional signage visible from the entrances to indicate location of stairs?			
5.6	Are stairs wide enough to accommodate groups of people travelling in both directions? **(Take a picture)**			
5.7	Are the stairs well-lit? (e.g., windows, lighting)			
5.8	Are the stairs clean?			
5.9	Is the temperature on the stairs too hot or too cold?			
5.10	Are the stairs well-decorated (e.g., art)?			
5.11	Are there any signs or marks promoting use of stairs? **(Take a picture)**			
5.12	Is there a gym? **(Take a picture)**			
5.13	Are there showers and/or changing facilities?			
5.14	Are there any other designated physical activity areas, apart from the gym (e.g., exercise room)? **(Take a picture)**			
5.15	Are there drinking water facilities or water fountains throughout the building? **(Take a picture)**			
5.16	Is water, tea, coffee provided to the staff at the working desk?			
5.17	Are there lunchrooms within 10 minutes of walking distance of workspaces?			
5.18	Are there canteens within walking distance of workspaces?			

General comments:

End time:

WORKSITE OBSERVATIONAL CHECKLIST
Assessment of Canteen

Name of Assessor: Date: Start Time:

a. How many canteens are there on the worksite premises? ______

Fill the form below for each canteen

Worksite:

Canteen type: ☐ Main ☐ Kiosk ☐ Other, specify ____________________________

Canteen Name (or Number):
Start Time:

		**Yes**	**No**	**Don't know**	**Comments**
**1**	**TYPES OF FOOD IN THE CANTEEN**				
1.1	Offers ONLY premade and pre-packaged options (cookies, cakes, or sandwiches) and the food is prepared off-site and brought in to the food service location				
1.2	Offers a small number of menu (less than 15) options including premade or quick to make options				
1.3	Offers a large number of menu (more than 15) options that are made in-house.				
1.4	The menu or food options offered in the canteen are changed at least once a week (comment on how often the menu is changed)				
1.5	A buffet is served				
1.6	(If yes to 1.5) A customer chooses the amount of food in the buffet				
**2**	**FOOD OPTIONS**				
**2.1**	**Are the following grains or grain products available?**				
2.1a	Whole grains (barley, brown rice, buckwheat, bulgur (cracked wheat), millet, oatmeal, popcorn, or whole-wheat bread, pasta, or crackers)				
2.1b	Refined grains (white flour, white rice and white bread, maize meal, pasta, or samp)				
**2.2**	**Are the following lentils or legumes available?**				
2.2a	Speckled beans				
2.2b	Kidney beans				
2.2c	Chickpeas				

2.2d	Soy				
2.2e	Peanuts and nuts				
2.2f	Other				
**2.3**	**Are the following vegetables available?**				
2.3a	Dark greens (broccoli, spinach, kale, or lettuce)				
2.3b	Orange and red vegetables (carrots, tomatoes, butternut/pumpkin, red pepper, or orange sweet potato)				
2.3c	Starchy vegetables (potato)				
2.3d	Other vegetables (celery, green beans, cauliflower, eggplant, cucumber, mushroom, peas, cabbage, beetroot, or pepper)				
**2.4**	**How are vegetables prepared and served?**				
2.4a	Raw				
2.4b	Steamed				
2.4c	Baked or grilled				
2.4d	Stir-fried				
2.4e	Deep-fried				
2.4f	Is oil visible on the cooked vegetable? (Take a picture)				
2.4g	Other				
**2.5**	**Are the following animal-based foods (non-vegetarian) options available?**				
2.5a	Eggs				
2.5b	Chicken				
2.5c	Fish				
2.5d	Mutton<br>(Specify, e.g., lamb)				
2.5e	Beef				
2.5f	Pork				
2.5g	Sea food (e.g., prawns, shrimps, lobsters)				
2.5h	Dried meat (biltong)				

2.5i	Processed Meats (Viennas/Polony, Russians, Boerewors sausage)				
2.5j	Other				
**2.6**	**How is meat prepared?**				
2.6a	Steamed				
2.6b	Baked or grilled				
2.6c	Stir-fried				
2.6d	Deep-fried				
2.6e	Curry				
2.6f	Is oil visible on the cooked meat? (Take a picture)				
2.6g	Other				
**2.7**	**Are the following fruit options available?**				
2.7a	Fresh whole fruits				
2.7b	Cut up				
2.7c	Frozen				
2.7d	Canned				
2.7e	Dried				
2.7f	Other				
**2.8**	**Are the following types of oil and fats available?**				
2.8a	Sunflower oil				
2.8b	Olive oil				
2.8c	Palm oil				
2.8d	Butter				
2.8d	Canola				
2.8e	Other				
**2.9**	**Are the following desserts available?**				
2.9a	Cake				
2.9b	Cookies				
2.9c	Ice cream				
2.9d	Other				

**2.10**	**Are the following Dairy products available?**				
2.10a	Yoghurt (low-fat, fat-free, etc.)				
2.10b	Maas (low-fat, fat-free, etc.)				
2.10c	Flavored milkshakes (low-fat, fat-free, etc.)				
2.10d	Milk (low-fat, fat-free, etc.)				
2.10e	All cheese				
2.10f	Other				
**2.11**	**Are the following beverages available?** [If available free of cost, please indicate in the comment section]				
2.11a	Regular soda				
2.11b	Diet soda				
2.11c	Fresh fruit juice				
2.11d	Canned fruit juice				
2.11e	Packaged juice				
2.11f	Water				
2.11g	Soda water				
2.11h	Other				
**2.12**	**Are the following types of milk available?**				
2.12a	Fat-free milk (Skimmed)				
2.12b	Low -fat milk				
2.12c	Whole milk (Full cream)				
2.12d	Other (Evaporated, buttermilk, condensed milk, or coconut milk)				
**2.13**	**Are the following types of tea and coffee available?**				
2.13a	Black tea				
2.13b	Milk tea				
2.13c	Rooibos tea				
2.13d	Black coffee				
2.13e	Milk coffee				
2.13f	Green tea (e.g. jasmine, chamomile)				

2.13g	Sugar is provided for customers to add (NOT added during preparation)				
2.13h	Other (e.g., iced tea, Milo, hot chocolate)				
**2.14**	**Is the following type of FREE and safe water available?**				
2.14a	Cold water				
2.14b	Hot water				
**3**	**PORTION SIZES**				
3.1	Is the sugar served separately from hot beverages?				
3.2	Is there more than one portion size option for food items? (e.g., 1/2 plate rice and a full plate rice; big can of soda and a smaller can of soda)				
3.3	Is a smaller portion size with proportional pricing available, for example, half-plate rice for half the price of the full plate?				
3.4	Is salt available on the dining tables?				
3.5	Is tomato sauce or sweet and spicy sauce available on the dining tables?				
3.6	Other				
**4**	**POINT OF CHOICE**				
4.1	Is there nutrition information on the kilojoules per serving provided on a large display or menu board?				
4.2	Is there nutrition information on the amount of salt/sodium in foods provided on a large display or menu board?				
4.3	Is there a system to identify healthier items in the canteen (e.g., icons or color codes for healthy and less healthy items)?				
4.4	Are price discounts for healthier foods (e.g., whole fruits) available?				
4.5	Are healthier foods (e.g., fruits, fresh fruit juice, diet drinks) placed closer to the customer?				
4.6	Are healthier foods (e.g., fruits, fresh fruit juice, diet drinks) placed at eye level?				
4.7	Are healthier food options promoted (e.g., through signs, banners, and/or kiosks)?				
4.8	Other				

**5**	**INFORMATION**				
5.1	Are there printed brochures with nutritional information (e.g., kilojoules/servings) at the entrance to the canteens on tables or elsewhere?				
5.2	Other				
**6**	**PICTURES AND DOCUMENTS**				
6.1	Copy of menu received				
6.2	Photo of canteen showing food options taken				
6.3	Brochures with nutritional information (If so, take one for reference)				

End time: ___________________

SUPPLEMENTARY FILE S3: Organisational Readiness for Implementing Change (ORIC) Questionnaire

Dear Participant,

The aim of the study is to reduce prediabetes among employees at your worksite through canteen improvements and lifestyle education. Small changes will be made to your worksite food environment for one year, for example, providing educational materials on healthy eating (posters, pamphlets, table tents), provision of free water and fruit, the introduction of brown rice, etcetera. The lifestyle education program will include goal setting to reduce weight, dietary education, stress education and physical activity classes. Staff will be invited to take part in the lifestyle education lessons. Before we design the intervention, we want to assess your worksite readiness to implement change.

By participating in this survey, you will be volunteering to contribute to the research study on the determination of the worksite readiness to implement change (Are employees willing to implement the changes at their worksite).Your assistance in the completion of the survey is highly appreciated. It would take approximately five minutes of your time. Your responses will be confidential - we will not collect identifying information such as your name, etcetera.

Your participation in this study is voluntary. If you decide to participate in this research survey, you may withdraw at any time. Should you wish to participate in this study, your answers will not be shared with anyone but will be used anonymously for only scholarly purposes by the research team.

Kindly choose your option according to the category mentioned below.

1	2	3	4	5
Disagree	Somewhat Disagree	Neither Agree nor Disagree	Somewhat Agree	Agree

Please indicate your position in the company (job title): ___________________________________

Please indicate which worksite you are employed at: ____

I am committed to implementing this change.	1	2	3	4	5
I am determined to implement this change.	1	2	3	4	5
I am motivated to implement this change.	1	2	3	4	5
I will do whatever it takes to implement this change.	1	2	3	4	5
I want to implement this change.	1	2	3	4	5
I feel this change is compatible with our values.	1	2	3	4	5
I need to implement this change.	1	2	3	4	5
I believe this change will benefit our worksite.	1	2	3	4	5
I believe it is necessary to make this change.	1	2	3	4	5
I believe this change will work.	1	2	3	4	5
I see this change as timely.	1	2	3	4	5
I believe this change is cost-effective.	1	2	3	4	5
I believe this change will make things better.	1	2	3	4	5
I feel that implementing this change is a good idea.	1	2	3	4	5
I value this change.	1	2	3	4	5
I know what it takes to implement this change.	1	2	3	4	5
I can keep the momentum going in implementing this change.	1	2	3	4	5
I can manage the politics of implementing this change.	1	2	3	4	5
I can support people as they adjust to this change.	1	2	3	4	5
I can get people invested in implementing this change.	1	2	3	4	5
I can coordinate tasks so that implementation goes smoothly.	1	2	3	4	5
I can keep track of progress in implementing this change.	1	2	3	4	5
I know how much time it will take to implement this change.	1	2	3	4	5
I know what resources we need to implement this change.	1	2	3	4	5
I know what each of us has to do to implement this change.	1	2	3	4	5
I have the equipment we need to implement this change.	1	2	3	4	5
I have the expertise to implement this change.	1	2	3	4	5
I have the time we need to implement this change.	1	2	3	4	5
I have the skills to implement this change.	1	2	3	4	5
I have the resources we need to implement this change.	1	2	3	4	5
The timing is good for implementing this change.	1	2	3	4	5

Thank you for your time!
